# Neurovasculature of high and low tie ligation of the inferior mesenteric artery

**DOI:** 10.1007/s00276-018-2092-3

**Published:** 2018-09-01

**Authors:** Amy Campbell, Angus Macdonald, Raymond Oliphant, David Russell, Quentin A. Fogg

**Affiliations:** 1 Laboratory of Human Anatomy, School of Life Sciences, University of Glasgow, Glasgow, UK; 2 Department of Surgery, Monklands District General Hospital, Monkscourt Avenue, Airdrie, North Lanarkshire ML6 0JS UK; 3 University of Dundee, School of Medicine, Ninewells Hospital and Medical School, Dundee, DD1 9SY UK

**Keywords:** High tie, Low tie, Inferior mesenteric artery, Neurovasculature, Histology

## Abstract

**Purpose:**

Controversy exists as to whether a high or low tie ligation of the inferior mesenteric artery (IMA) is the preferred technique in surgeries of the left colon and rectum. This study aims to contribute to the discussion as to which is the more beneficial technique by investigating the neurovasculature at each site.

**Methods:**

Ten embalmed cadaveric donors underwent division of the inferior mesenteric artery at the level of the low tie. The artery was subsequently ligated at the root to render a section of tissue for histological analysis of the proximal (high tie), mid and distal (low tie) segments.

**Results:**

Ganglia observed in the proximal end of seven specimens in the sample imply that there would be disruption to the innervation in a high tie procedure.

**Conclusion:**

This study suggests that a high tie should be avoided if the low tie is oncologically viable.

## Introduction

The inferior mesenteric artery (IMA) has been the centre of great debate in the world of colorectal cancer surgery since two opposing techniques for resection were proposed over 100 years ago [1, 2]. Despite a large volume of academic research investigating a variety of relevant aspects including anastomotic perfusion [3], autonomic innervation [4] and long-term morbidity and mortality rates [5], controversy still exists as to which is the preferred technique; currently there is wide variation in surgical practice.

Surgery that includes high tie of the IMA is generally considered to have taken place when the IMA is resected at its origin with the abdominal aorta. However, this is the cause of some debate as a minority of the surgical literature refer to high tie as ‘about 1 cm from its [the IMA] origin’ [6]. For the purpose of this study, high tie is considered to be the ligation of the IMA at the origin with the aorta i.e., flush with the aorta. Proponents of this technique have argued that it is more beneficial from an oncological perspective as it ensures complete resection of all malignant lymph nodes around the IMA and allows improved accuracy of tumour staging [7, 8]. Other reported advantages include improved 5-year survival [9], though this has been disputed [5]. Population-based studies have also shown that there is no increased risk of anastomosis leakage following high tie ligation [10], though this does not appear to be true for those patients with increased cardiovascular risk factors [11].

Similarly, there is conflicting classifications of the low tie ligation ranging from ‘the level of the superior rectal artery’ [12] to simply ‘below the origin of the left colic artery’ [5]. In fact, significant and frequent anatomical variation in the division of the IMA has been evidenced which may be part of the reason a consensus has not been reached on definition [13]. This study goes on to argue that the anatomical variation discovered is evidence that high tie should be considered the ‘only relevant procedure’ in cases of colorectal neoplasm where it is vital to ensure complete lymphadenectomy.

For the purpose of this study, low tie is considered to be at the level of the superior rectal artery, just distal to the origin of the left colic artery. Advocates of the low tie technique state that it carries an understandable reduction in intra- and post-operative complications by ligating a distance away from the abdominal aorta [6]. However other findings suggest that the low tie procedure causes more blood loss to the patient, on average, despite reduced operating times [14]. With regards to perfusion, the low tie is argued to result in a significant increase in blood flow ratio supplying the anastomosis. This is due to blood being supplied through both the marginal artery (of Drummond) and the left colic artery in a low tie, while with the high tie the anastomosis is completely reliant on the marginal artery (of Drummond) alone [12]. These findings are consistent with a recent publication which states that high tie ligation may be related to proximal bowel necrosis [15].

Further considerations of this study include the sacrifice of autonomic nerves in high and low tie procedures. Based on current anatomical understanding these are believed to be of the superior hypogastric sympathetic plexus which, when damaged due to atherosclerotic disease, have been found to cause impotence in 90% of males [16]. More recently, these have been found to be related not only to sexual function but to urinary incontinence in surgery for rectal cancers [17]. A 2006 study found that, with regards to surgical preservation of autonomic nerves, the origin is the only safe site for ligation [4], but many published works disagree with this finding [18–20]. Establishing the current level of relevant knowledge on the anatomy and innervation of this plexus and the nerves that it projects can be used as a base upon which further investigations can be built.

Therefore, the aim of this study is to describe the neurovasculature at the levels of the high and low tie ligations.

## Materials and methods

**Fig. 1:**
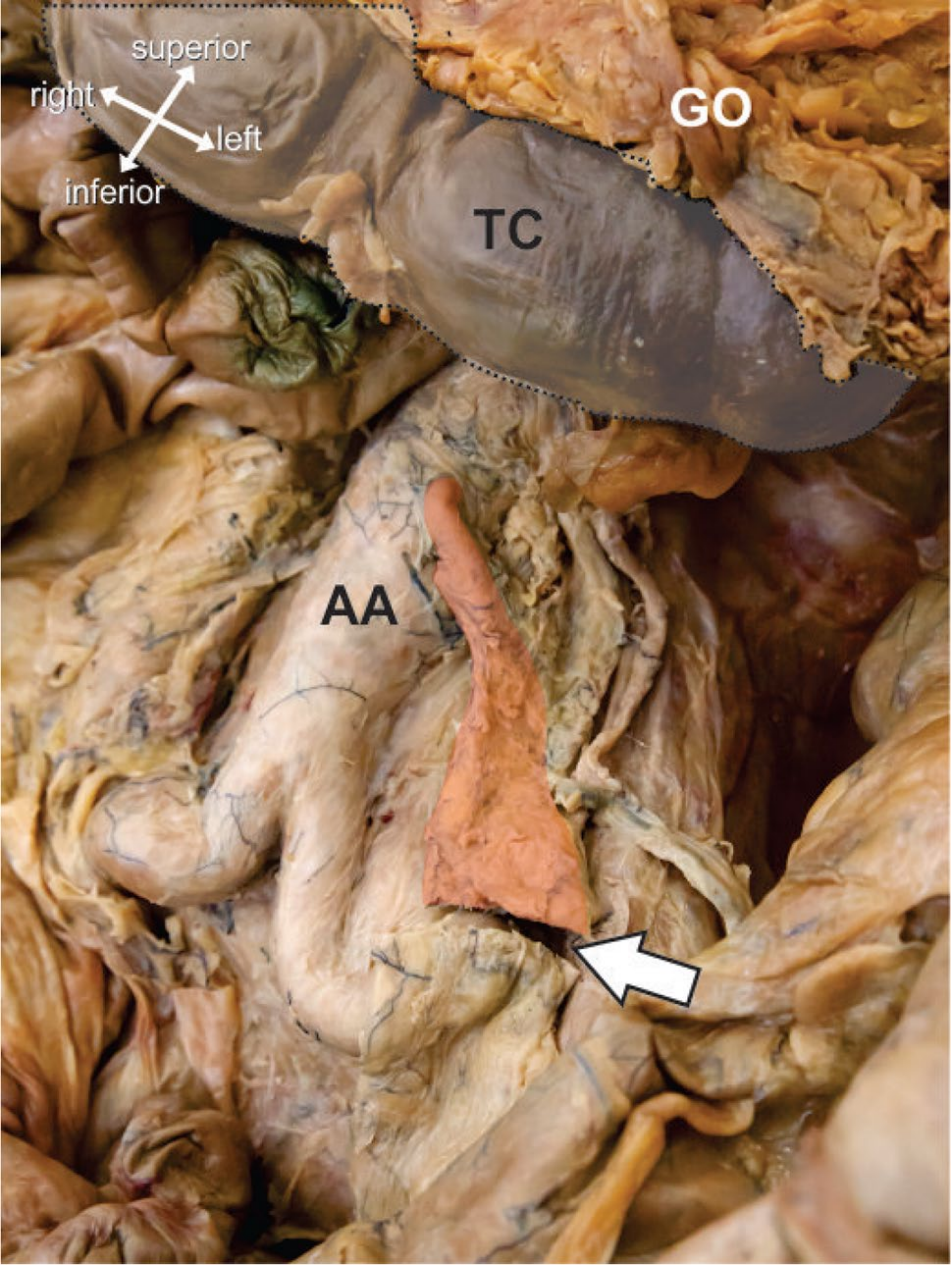
Anterolateral view of the abdomen. The greater omentum (GO) and transverse colon (TC) have been retracted superiorly. The mesentery (not shown) and posterior parietal peritoneum (PP) have been reflected laterally. The inferior mesenteric artery and supporting tissues (red) was isolated from its branching point from the abdominal aorta (AA), sectioned at a level appropriate for a low tie (arrow). (Color figure online)

Twelve embalmed cadaveric donors (male *n* = 4, female *n* = 8; mean age 84.8 years) were the subject of the current study. Ethical approval for the project was granted by the Lead Licence Holder, under the terms of the Anatomy Act (1984) and the Human Tissue (Scotland) Act 2006. Two donors were immediately excluded due to previous abdominal laparoscopic surgery deforming the anatomy in the region of the IMA. The abdomen was opened and the IMA located and divided at putative level of low tie by a local consultant colorectal surgeon and surgical registrar experienced in colorectal surgery (now also a consultant colorectal surgeon). Blunt dissection of abdominal fascia and mesorectum exposed the bifurcation of the abdominal aorta and, superiorly, the origin and branches of the IMA (Fig. 1). Photographs of each specimen were taken with a digital camera (Canon EOS 5D, Japan).

**Fig. 2:**
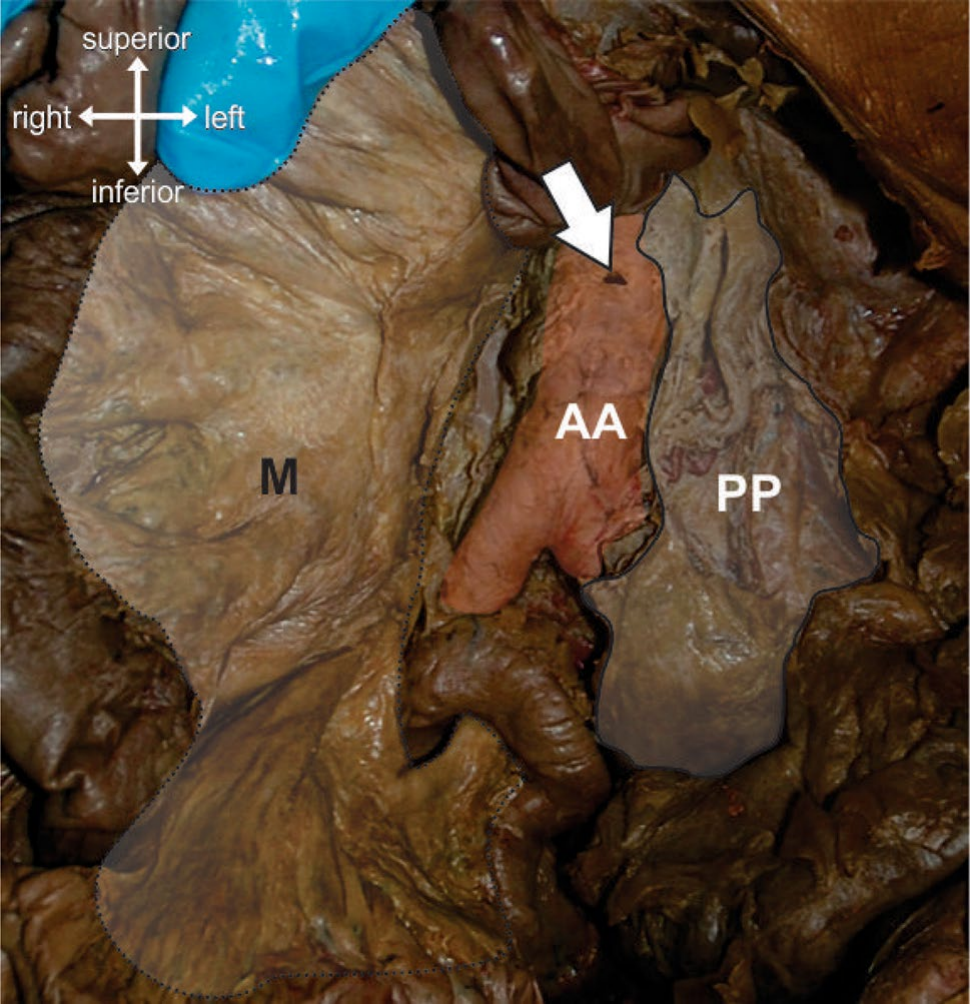
Anterior view of the abdomen. The mesentery (M) has been reflected laterally and the posterior parietal peritoneum (PP) sectioned to reveal the abdominal aorta (AA). The inferior mesenteric artery has been removed from its branching point (arrow). (Color figure online)

In each cadaver, the IMA was removed at origin (Fig. 2) and photographs were repeated. The remaining tissue was then divided into 10 mm sections. Wax embedding of these sections from distal to proximal was followed by microtome sectioning, staining with Masson’s Trichrome and analysis using a light microscope (Zeiss Axioskop, Germany) and digital capture software (Zeiss Axiovision, Germany).

## Results

**Fig. 3:**
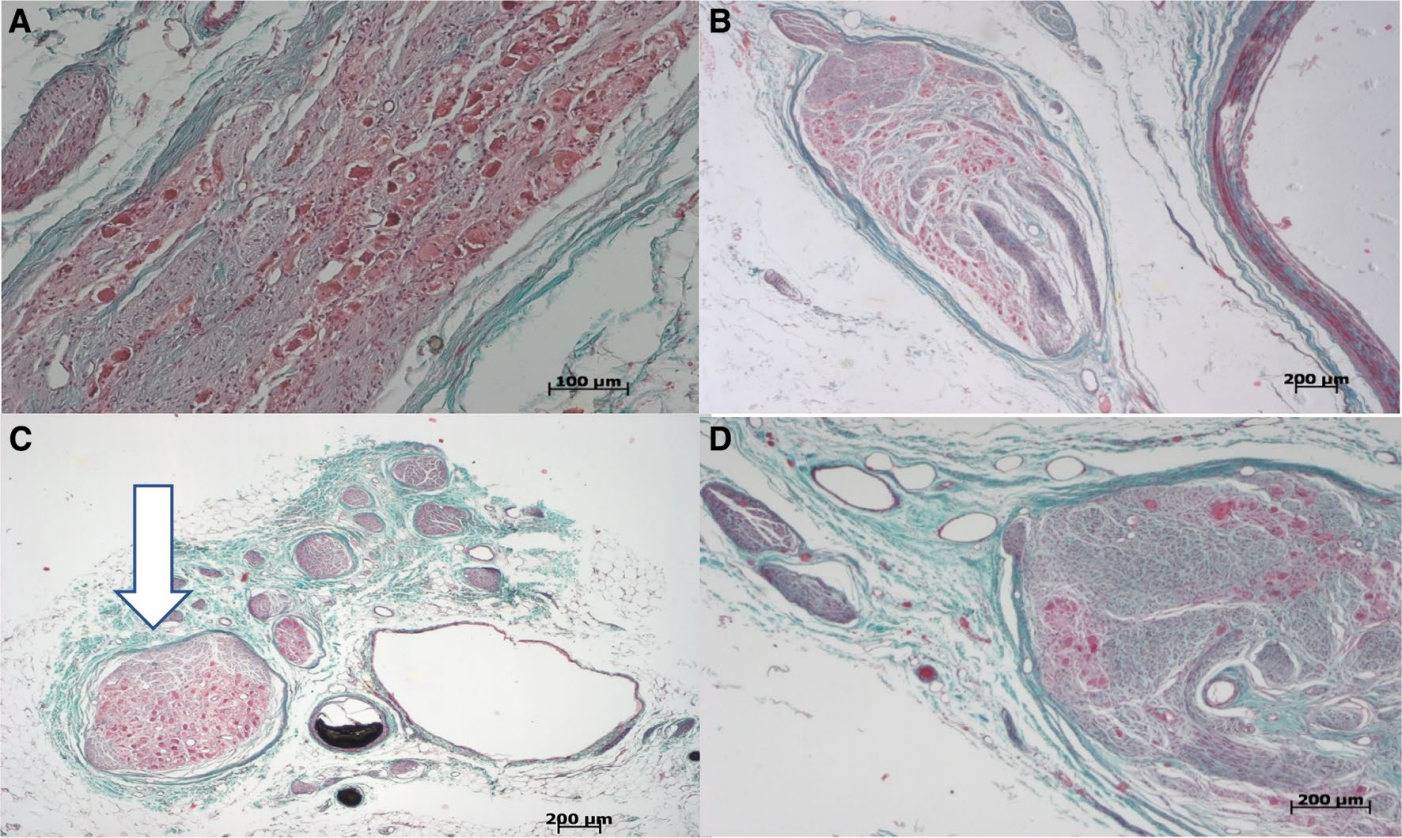
Ganglia observed at proximal portion of IMA in a selection of specimens. **a** Specimen number 1 demonstrating ganglia at proximal portion of IMA, **b** specimen number 2 demonstrating ganglia at proximal portion of IMA, **c** specimen number 3, demonstrating ganglia at proximal portion of IMA comparable in size to adjacent inferior mesenteric vein (arrow); **d** specimen number 4, demonstrating ganglia at proximal portion of IMA. Masson’s Trichrome. (Color figure online)

During histological analysis, a large volume of autonomic ganglia were noted at the proximal end of seven specimens (70%; Fig. 3) which can be considered to have been sacrificed in high tie ligation.

**Fig. 4:**
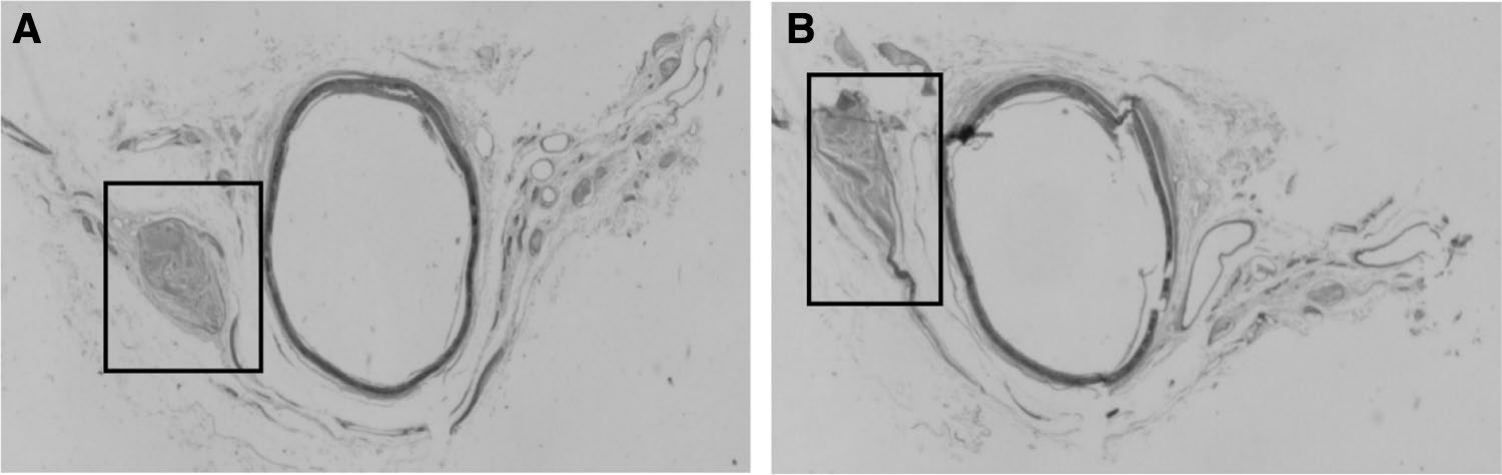
Ganglia observed in IMA sections of specimen 2. **a** Mid section, **b** distal section

The ganglia noted in one specimen (10%) were comparable in size to the inferior mesenteric vein. In the mid and distal section of eight specimens (80%), the nerve distribution was found to be that of the *nervi vascularis*, which are the circumferential nerves around the blood vessels. This was not true, however, in one specimen (10%), as large ganglia were found in both the mid and distal sections (Fig. 4).

The respective length of each specimen is detailed in Table 1. The length of removed specimens ranged from 1.4 to 6.8 cm with a mean length of 3.4 cm.

**Table 1:** Respective length of each specimen taken from the root of the abdominal aorta to level of low tie

Specimen number	1	2	3	4	5	6	7	8	9	10
Length	6.8 cm	3.9 cm	2.0 cm	1.4 cm	2.35 cm	4.4 cm	3.6 cm	2.4 cm	2.6 cm	4.7 cm

## Discussion

A number of published studies argue that the main benefit of ligating the IMA at the origin is that this ensures complete resection of malignant tissue [3, 8]. In the current study, no noticeable nodular lymph tissue over and above the lymphatics was observed throughout histological analysis of the neurovasculature. This is most likely due to the fact that the sample population did not have colorectal disease and as a result enlarged nodular lymph tissue was not observed in the neurovasculature of the IMA. Further studies may wish to look at individuals, such as those excluded in the current study, who have been diagnosed with colorectal cancer. In these studies, the lymph tissue in the region between the high and low tie may be quantified and compared against a control sample of individuals who do not have any colorectal malignancy. As such, further investigation into whether or not the high tie is in fact the most beneficial technique from an oncologically viable perspective may be carried out.

The median age of the sample population (89.5 years) and that of a patient undergoing colorectal cancer surgery in the local area (69.1 years) [21] are similar enough that the sample population can be considered realistic. The elderly age of patients at diagnosis also raises some concerns with regards to co-morbidities, such as disease of the arteries, which may result in the anatomy of the vasculature being disrupted. Patients with severe stenosis or abdominal aortic occlusion may result in lower limbs being supplied by a collateral circulation from the IMA and its branches without which, irreversible lower limb ischaemia can occur [22]. However, no evidence of this was found in the present study.

The current study suggests that when considering the neurovasculature, the low tie is the preferred method in order that the autonomic ganglia observed are not unnecessarily sacrificed in cases where there is not obvious lymph involvement in and around the IMA. The discovery of unconvincing *nervi vascularis* in the mid and distal section of one specimen may be attributed to the fact that the individual in question had had a previous tumour in the upper abdomen which had caused the aortic bifurcation and the surrounding anatomy to become orientated to the right and as a result the anatomy was not what was expected in an average individual. The decision was taken to include this donor as, unlike in the two excluded specimens, the region of the IMA was unaffected by the superior growth. Similarly, the section taken for histology sampling from another donor was much shorter (1.4 cm compared to a 4.35 cm average of the other specimens). This could be attributed simply to surgical and anatomical variation. Damage to these ganglia, which act as junctions between the superior hypogastric plexus and the inferior hypogastric plexus supplying the abdominal and pelvic viscera, may be associated with visceral pain. However, there is currently no literature which suggests that this is a common complaint following colorectal resection. The supply of the hypogastric plexus to the pelvic viscera seems to be the cause of more interesting complications of nerve damage after surgery. Damage to this nerve plexus has been associated with erectile dysfunction in males [23]. Given the median age of patients at diagnosis is over 70 years [24] these individuals are perhaps less likely to report issues concerning sexual difficulties. In surgeries for benign disease, such as diverticular disease or inflammatory bowel disease, which typically present in males at a much younger age (29.5 years in Crohn’s Disease) [25], the IMA is not divided and the tie is taken much further from the abdominal aorta to avoid severing nerves which could damage sexual function, this is evidenced by findings that 11% of men undergoing surgery to treat inflammatory bowel disease report sexual dysfunction in comparison to 50% of subjects being treated for cancer [23]. The pelvic splanchnic nerves, which are responsible for urinary continence and erectile function at vertebral levels S2–S4, track anteriorly and laterally to join the inferior hypogastric plexus anteriorly. This union occurs lower than the level of the origin of the IMA and it is unlikely that these nerves are damaged outright in high tie ligation despite studies which suggest that urinary incontinence and erectile dysfunction are affected together or not at all [17]. Operatively, these nerves are more likely to be damaged during rectal mobilisation in the pelvis and this would occur in either high or low tie patients. Meanwhile female sexual function, and indeed dysfunction, being a less visually apparent failure, has resulted in poor classification in the literature and anecdotal stories from surgeons which suggest that women are much less likely to return with complaints of this nature in clinics.

The authors recognise the limitations of this study in relation to its anatomical correctness. However, as we know now that overall survival is dependent, as much on co-morbidity (cardiac, renal and respiratory function) as on issues of quality of life which are gaining a greater profile. Our study could be repeated in the clinical/operative setting where an accurate record of anatomy, pathology and function could be undertaken.

The fact that over 16% of the original sample population had to be excluded due to previous intestinal surgery highlights the importance of the research into finding a protocol for what is an increasingly common surgery. In comparison to much of the literature, this study was multifactorial and considered not only the vasculature but also the relevant innervations. The current study supports the low tie technique in oncologically appropriate cases as discovery of ganglia at the root of the IMA highlights the sacrifice of autonomic innervations with high tie ligation. It has been suggested that the debate on the level of ligation of the IMA will not be resolved until, amongst others, the preoperative anatomy can be properly evaluated [26]. The current study helps to progress this debate not least because it highlights a clear anatomical variation in the length of the inferior mesenteric artery from high to low tie between specimens which, as described, ranged from 1.4 to 6.8 cm. To date, this is the first study to highlight this anatomical variation and future work investigating how this may relate to the quantity of lymph nodes in the area and subsequent disease outcomes, would be of interest.
